# An historical overview of the National Network of Libraries of Medicine, 1985–2015

**DOI:** 10.5195/jmla.2018.297

**Published:** 2018-04-01

**Authors:** Susan L. Speaker

**Affiliations:** History of Medicine Division, National Library of Medicine, Bethesda, MD

## Abstract

The National Network of Libraries of Medicine (NNLM), established as the Regional Medical Library Program in 1965, has a rich and remarkable history. The network’s first twenty years were documented in a detailed 1987 history by Alison Bunting, AHIP, FMLA. This article traces the major trends in the network’s development since then: reconceiving the Regional Medical Library staff as a “field force” for developing, marketing, and distributing a growing number of National Library of Medicine (NLM) products and services; subsequent expansion of outreach to health professionals who are unaffiliated with academic medical centers, particularly those in public health; the advent of the Internet during the 1990s, which brought the migration of NLM and NNLM resources and services to the World Wide Web, and a mandate to encourage and facilitate Internet connectivity in the network; and the further expansion of the NLM and NNLM mission to include providing consumer health resources to satisfy growing public demand. The concluding section discusses the many challenges that NNLM staff faced as they transformed the network from a system that served mainly academic medical researchers to a larger, denser organization that offers health information resources to everyone.

The Regional Medical Library (RML) Program, now the National Network of Libraries of Medicine (NNLM), has a rich and remarkable history, beginning in 1965. Alison Bunting, AHIP, FMLA, ably documented the network’s first twenty years in her 1987 article, “The Nation’s Health Information Network: History of the Regional Medical Library Program, 1965–1985” [1].

The following historical overview traces the major trends in the network’s development between 1985 and 2015, drawing on three decades of primary and secondary sources. These include the library periodical literature, RML directors’ meetings minutes, statements of work for successive five-year RML contracts, National Library of Medicine (NLM) annual reports, RML newsletters and blogs, and other materials, along with informal conversations with National Network Coordinating Office staff members. A careful review of these sources reveals how the network transformed itself during this time: growing, adapting, and evolving as information technology came of age and as both NLM and the RMLs, which originally focused on serving academically affiliated physicians, came to support a much larger, denser network of organizations offering health information resources to everyone.

## REGIONAL MEDICAL LIBRARY (RML) DEVELOPMENT TO 1985

The idea for regional medical libraries emerged in the early 1960s, as medical school librarians, physicians, and federal officials began to consider how to improve the often poor condition and inadequate collections of US medical school libraries. In 1964, the President’s Commission on Heart Disease, Cancer and Stroke noted that communication of current research would be essential in the battle against those diseases and warned that “unless major attention is directed to improvement of our national medical library base, the continued and accelerated generation of scientific knowledge will become increasingly an exercise in futility” [1].

The Medical Library Assistance Act of 1965 [2], part of a deluge of health-related legislation enacted that year, authorized NLM to provide grant funding to improve the condition and capabilities of medical libraries throughout the United States. The grants were available for (1) construction of new and renovation, expansion, or rehabilitation of existing medical library facilities; (2) training of medical librarians and other information specialists in the health sciences; (3) assistance to special scientific projects; (4) research in the field of medical library science and related fields; (5) improvement and expansion of basic resources of medical libraries and related facilities; (6) development of a national system of regional medical libraries; and (7) preparation of biomedical scientific publications [1].

During the next five years, NLM worked with medical libraries around the country to delineate the geographical regions and decide which institutions should be designated as the RML for each one. The first RML, Harvard University’s Countway Medical Library, became operational in 1967; another ten were designated by 1970. Within each of the original eleven geographical regions was an RML; a number of Resource Libraries, usually medical school libraries; and a larger number of smaller Basic Health Sciences Libraries, located in hospitals, medical societies, and so on. Initially funded by grants, the RML network’s basic activities were supported with competitive three-year contracts (later increased to five years) from 1971 to 2016. In 1982, with the Reagan-era government budget cuts, the network was reconfigured into seven regions.

The core responsibilities of the RMLs in the early years were to: (1) provide health professionals with basic information services: access to books, journals, audiovisuals, and literature databases; (2) improve and extend the interlibrary loan (ILL) services; (3) coordinate and extend the regional network; (4) coordinate the collection of regional holdings data; (5) compile union lists (which contributed to NLM’s Serials Holdings database and facilitated ILL work); (6) train regional library staff to use NLM’s medical citation database (the Medical Literature Analysis and Retrieval System [MEDLARS]) and other computer-based resources as they were developed; (7) assess the region’s information needs and conduct evaluations of the various RML projects; and (8) promote the RMLs’ work via published articles and newsletters about RML activities and exhibits at professional meetings. These duties have remained basic to the RML mission since then.

By 1985, the RML network included over 3,000 libraries in 50 states and US territories. Previously inadequate libraries had expanded and improved their facilities, and all the network members had strengthened their collections of books and journals. Likewise, many librarians had received extensive training, which helped them do much more for both patrons and colleagues. The network had greatly improved delivery of documents to health professionals through its ILL network. NLM’s new DOCLINE, an automated ILL referral and routing system, would soon transform the ILL process, radically reducing the time that network librarians and staff spent on document requests and freeing up time for other activities. NLM, with two of the RML regions, had conducted a pilot test of electronic mail for submitting ILL requests and developed a format for email requests. The network libraries were also using telefacsimile (“fax”) technology for rapid document transfer in response to emergency requests.

Access to the MEDLARS medical citation database had expanded enormously. In the early years, library patrons had to submit their search requests to specialists at NLM (or later, at RMLs); the requests were processed by MEDLARS in batches; and results returned in 3 to 4 weeks. Available through just 3 search centers in 1964, the database could be accessed directly by over 4,000 libraries and individuals in 1985, through MEDLINE (“MEDLARS Online”). And GRATEFUL MED, a desktop computer application that would allow health professionals to search MEDLINE independently, was in final testing and would be introduced the next year.

The basic goals for the 1986 to 1991 RML contracts, which followed the recommendations of a 1984 NLM Working Group, were similar to those of previous years: improve access to and delivery of information to health professionals, develop and maintain an effective and efficient network of health sciences libraries, and develop and maintain linkages between the network and other library/information networks or health professional organizations to share resources [1]. As the new contracts emphasized providing information services regardless of geographic location, each RML identified underserved areas in its region and developed plans to improve access. Network members continued to evaluate how well network goals were met, assess the benefits of network services, and examine new or emerging information needs. A new element in that contract cycle was funding for cooperative acquisitions of most types of library materials, on a regional basis.

The 1986 to 1991 contracts stipulated that the seven RMLs would continue to “coordinate network activities in areas such as interlibrary loan, development and maintenance of union lists, and cooperative acquisitions and resource sharing programs.” While the RMLs would continue to coordinate educational and training programs to some extent, consulting and training programs for hospital library managers would be phased out. Educational or consulting needs would be handled by regional consultants. Likewise, online training and services would be handled by three of the RMLs: the New York Academy of Medicine, the University of Nebraska, and the University of California, Los Angeles (UCLA) Biomedical Library.

The RML network would also participate in two new programs: a national reference referral network and a national preservation plan for medical libraries [1]. The contract “Statement of Work” also placed more emphasis on cooperating with NLM in testing new products and services, such as GRATEFUL MED, the desktop computer software for independent MEDLINE searching. In the late 1980s, the RMLs helped NLM with two important research and evaluation projects, one that assessed MEDLINE available on CD-ROM and another investigating physicians’ use of MEDLINE for clinical problem solving [3, 4].

## RECONCEPTION OF THE RMLS

At this point, NLM and its RMLs were on the cusp of several major changes. The first was a shift in their basic mission, as outreach became a priority in the late 1980s. The second was the advent of the Internet during the 1990s, the migration of NLM and RML resources and services to the World Wide Web, and a mandate to encourage and facilitate Internet connectivity in the RML network. The third was the further expansion of the NLM/RML mission to include consumer health information resources to satisfy growing public demand. The basic RML network structure and its core responsibilities would provide a good foundation for the expansion that followed, although growing pains would be part of the process as well.

In 1987, the Senate Appropriations Committee for the Departments of Labor, Health and Human Services, and Education lauded the accomplishments of NLM and the RML Program, but noted that many health professionals were still unaffiliated with a health sciences library and so did not have ready access to the vital health information that they needed. Congress urged NLM to develop an active outreach program to raise awareness of NLM resources and enable access for *all* health professionals—physicians, nurses, midwives, psychologists, and others—especially those working in rural or inner city areas. They also amended the National Library of Medicine Act, adding “Publicize the availability of [its] products and services” to NLM’s basic functions [5].

Responding to this mandate, the NLM Board of Regents convened an outreach planning panel, chaired by Dr. Michael E. DeBakey, to outline a strategy for ensuring that American health professionals were aware of how NLM’s information services could be used to assist in biomedical research, education, and practice. The panel members realized early on that the libraries of the RML network were essential for increasing information access for health professionals. The panel’s 1989 report, “Improving Health Professionals’ Access to Information: Challenges and Opportunities for the National Library of Medicine,” recommended, among other things, that:

NLM and the RMLs should build a more active partnership for the RML network, one that will be flexible and permit rapid response to regional needs, geographic factors, and changing environmental conditions. The emphasis of the RML Program should be to bring biomedical information resources within easy reach of all health professionals, especially those individuals in areas that do not currently have direct access. To do this, the RMLs should act as a “field force” for NLM products and services, providing information and services to health professionals directly through network libraries, and providing feedback from health professionals to NLM. [6]

The panel’s recommendations rapidly led to changes in the mission, goals, configuration, and even the name of the network. In 1990, it became the National Network of Libraries of Medicine (NNLM) to reflect its national structure and direction (supplemental Appendixes A and B). Within NNLM, the lead institutions that were under contract to NLM to provide network services would continue to be referred to as RMLs; the major medical libraries that served as important state or subregional service points would continue to be called Resource Libraries. But those local institutions formerly called Basic Health Sciences Libraries would be called Local Libraries, soon changed to Primary Access Libraries (PALs). Meanwhile, the network added an eighth RML when the ten-state Greater Northeastern Region (Region 1) was divided into two smaller regions—Regions 1 (Middle Atlantic Region) and 8 (New England Region)—to enable more intensive outreach efforts in this area.

According to the revised RML mission statement, “The mission of the National Network of Libraries of Medicine (NNLM) is to provide equal access to biomedical information to all U.S. health professionals in order to advance the progress of medicine and improve the public health” [7]. The network’s new goals were (1) to promote awareness of and access to biomedical information resources for health professionals, and (2) to develop and improve the biomedical information resources in the regions and support the sharing of these resources in the regions and throughout the nation.

NLM’s contract for the RML five-year period starting in 1991 stated that NLM and RMLs would be creating a “new” national program that incorporated the individual health practitioner in the institutional network by involving all the libraries in the network in establishing direct contact with health professionals. The new program would offer high-quality products and services to meet health professionals’ information needs, particularly those individuals with no library affiliation, efficiently and at reasonable cost. Further,

*In this new alliance, the RMLs and network libraries are looked to as representatives and agents for NLM information products and services, insofar as possible. Under the leadership of NLM, the RMLs are expected to assist NLM in developing products and services and in serving as a “field force” for marketing and distributing them. The RMLs are also expected to supply NLM with feedback concerning how information is being used, suggestions for improvements of existing products and services, new ideas for products and services, etc.* In all of these areas, the RMLs are expected to involve other libraries in the network to the greatest extent possible. Programs should be designed to facilitate communication among NLM and network libraries as well as from NLM through the RMLs and network libraries to health professionals, and back the other way. [7] [emphasis added]

## OUTREACH TO MORE HEALTH PROFESSIONALS

Intensive outreach work was already underway by the time these new contract requirements were published. Between 1989 and 1994, NLM, the RMLs, and NNLM member libraries made great strides in connecting more health professionals with information resources. A review of NLM’s outreach activities during this period noted the striking changes in network use statistics. For example, in 1989, there were 16 outreach projects underway in 14 states and the District of Columbia. In 1994, close to 300 outreach efforts had been carried out, involving every state, the District of Columbia, and the US Virgin Islands, reaching more than 500 institutions. In 1989, there were approximately 30,000 MEDLINE access codes issued throughout the United States; by the end of 1994, there were 100,000. In 1989, there were approximately 4 million searches conducted on NLM databases; in 1994, the number of searches was close to 7 million. In 1989, GRATEFUL MED users represented less than one-half of the total number of users each month; 5 years later, more than three-fourths of the searchers of NLM’s databases used GRATEFUL MED. Similarly, in 1989, GRATEFUL MED users accounted for less than one-third of the search sessions conducted on NLM’s system; by 1994, they accounted for two-thirds of the search sessions. Much of this progress could be credited to the RMLs’ many efforts to make health professionals aware of NLM’s resources, to facilitate access, and to provide training in the use of GRATEFUL MED [8].

Between 1989 and 1994, each RML developed projects that specifically focused on reaching unaffiliated (i.e., not affiliated with an institution with a medical library), rural, and minority health professionals, and providing GRATEFUL MED training and support so that these individuals could access the medical literature from any location. All RMLs conducted a minimum of twenty GRATEFUL MED training sessions per contract year for underserved professionals. An outreach coordinator or education coordinator (or both) for each RML traveled throughout the region demonstrating GRATEFUL MED and introducing unaffiliated health professionals to NNLM and NLM services. Besides this, network libraries might assist the RML with outreach efforts through subcontracts. Each RML determined the approach it used to carry out its outreach objectives, tailoring efforts to the region’s particular population and needs.

Outreach activities targeted a number of different categories of health professionals, including physicians, nurses, dentists, pharmacists, therapists, and health administrators. In some cases, RMLs focused on specific groups, for example, nurses, professionals serving mainly minority populations, or those working in rural areas or community-based organizations that provided support services to populations affected by HIV/AIDS [8]. RML staff also worked at times with Area Health Education Centers, which facilitated professional training and continuing medical education opportunities in underserved areas.

Realizing that promoting NLM products and services to new audiences effectively might require more sales and communications skills, the MidContinental (Region 4) RML staff used part of their budget to carry out surveys and focus groups to develop a marketing communications plan for their region. They queried a variety of health professionals about their familiarity with NLM and its products and services, primary sources of information used, primary uses of information, barriers to using NLM resources, the ideal information system, and preferred means of communication from NLM and the RML. From these data, they developed a detailed plan for communicating with network librarians, increasing awareness of NLM and RML services, and obtaining feedback on those services [8–10]. Other RMLs also made greater use of focus groups as time went on, with generally good results.

Exhibits were also an important means of promoting NLM’s products and services. By 1996, the RMLs were responsible for 60%–65% of the 60–70 exhibits and demonstrations of NLM’s products and services provided at health professional meetings each year. With the participation and involvement of the NNLM libraries, the number of exhibits held each year had increased fourfold, including more state, regional, and local meetings as well as national meetings. Most of the exhibits showcased NLM’s databases (primarily MEDLINE) and GRATEFUL MED, but resources produced by Specialized Information Services, the National Center for Biotechnology Information, the Lister Hill Center, and other NLM products were also featured. During this period, NLM also carried on many outreach activities that did not involve the RMLs directly, including publicity campaigns, a 1992 two-part satellite broadcast symposium on information resources and services available through hospital libraries (“Information STAT! Rx for Hospital Quality”), and HIV/AIDS outreach [8].

## ARRIVAL OF THE INTERNET

The mission and work of NLM and NNLM were also profoundly affected during the 1990s by the growth of the Internet. The federal High Performance Computing and Communications (HPCC) initiative began in 1992 (with multiyear funding authorized by the High Performance Computing Act of 1991 [11]). The HPCC was “a research and development effort with the goals of creating computers a thousand times faster than today’s supercomputers and a National Research and Education Network (NREN) that transmits information at speeds one hundred times faster than today’s wide-area computer networks” [12]. The initiative had four major components: High Performance Computing Systems, Advanced Software Technology and Algorithms, National Research and Education Network, and Basic Research and Human Resources.

NLM was the first institution in the Department of Health and Human Services to create an HPCC program. In its first year (1992), it supported advanced technology and systems such as the enhancement of the Unified Medical Language System and the 3-D anatomical imaging of the Visible Human project, and a Medical Connections grant program to provide “jump-start” funding to medical schools, hospital libraries, or practice groups wishing to connect to the Internet or expand regional network connections. NLM launched its first website in 1993 and, within several years, would be able to offer many of its resources via the Internet, including the NNLM website, which debuted in January 1995.

NLM quickly enlisted the librarians of NNLM in this new initiative. They were already experienced database users, and many were already using electronic mail, email discussion lists, telnet services, and other tools. And they had for years been assessing the nature and needs of medical libraries in their regions. In 1992, the Pacific Northwest RML at the University of Washington (Region 6) was awarded an enhancement to its contract to explore the efficacy of Internet connections and resources in community hospitals. The “Bench to Bedside” project extended Internet connectivity to seven Pacific Northwest community hospital libraries, several in remote areas, and provided the RML staff with many basic lessons in the process [13].

Other RMLs also initiated projects to get member libraries online, providing classes that introduced staff to the Internet and locating affordable Internet service providers. Meanwhile, the NNLM National Network Office (now the National Network Coordinating Office) had been working on several projects to obtain Internet access for the PALs: an interagency agreement with NASA to use their Internet data and telecommunications infrastructure, including access to LIFENET, and guest or “courtesy” Internet accounts at institutions that were already Internet sites.

These early efforts paid off. In the fall of 1993, the RMLs did a brief survey to assess the status of Internet connections among the 4,000 member libraries. Of the 3,331 responding libraries, 34% overall had Internet access. Among academic health sciences libraries (16% of those responding), 72% had access. Among hospital libraries (63% of those responding), only 24% had access. The primary use of the Internet, in those that had access, was for email, with searching remote databases secondary to that [14]. By 1999, a follow-up survey of Internet connectivity found that 100% of the academic medical libraries, 91% of hospital libraries, and 97% of other medical libraries had connections [15].

Internet connection work was formally incorporated into the RML contract for 1996–2001, which listed among the NNLM goals: “To encourage, develop, and support connectivity to the Internet and the inclusion of network member libraries and health professionals in the developing national information infrastructure.” A new outreach component, “Applied Technology” (including “Internet” and “Technology Awareness”) was added, appearing ahead of “Direct Outreach to Health Professionals” and “Exhibits” [16]. The RMLs were directed to assist the PALs to obtain connections, train librarians to use the Internet and online resources, develop Internet training materials to post online, publish fact sheets, articles, and so on to keep librarians updated about the Internet. Likewise, they were to provide “train the trainers” classes so librarians could train other librarians and health professionals in their regions.

Outreach to health professionals also included new components: developing programs focused on special populations or subject disciplines that had been identified as priority initiatives, for example, AIDS and senior health; developing projects with one or two inner city institutions; and identifying institutions that train minority health practitioners or serve minority populations and ensuring that they can access the network. Finally, the RMLs were to start developing pilot programs to provide consumer health information.

## HEALTH INFORMATION FOR EVERYONE

The 1990s brought a growing demand for consumer health information, as “managed care” medical insurance organizations became more common and health care reform debates went on. Better access to health information was seen as a good strategy for reducing costs and increasing patient satisfaction. Federal programs, such as the Healthy People initiatives, were also focusing on consumer access to health information as a way to reduce health disparities.

NLM began working on a consumer information clearinghouse focused on women’s health topics in 1994. Several of the RMLs had begun HIV/AIDS outreach projects, and NLM offered its AIDS databases free online in 1995. GRATEFUL MED made its Internet debut in 1996. MEDLINE was offered free on the web via PubMed the following year, resulting in an explosive increase in MEDLINE searches (from 7 million to over 220 million per year by 2000). Many of the new users (about one-third) were not health care professionals, but members of the general public, seeking health information for themselves and their family members. NLM responded to this trend with more focus on improving web access and developing more consumer-friendly databases, along with doing research on interface and connectivity problems.

Several surveys of library use suggested that public libraries were increasingly providing Internet access for consumers looking for information, including health information [17], so NLM carried out a one-year pilot project “to learn about the role of public libraries in providing health information to the public and to generate information that would assist NLM and the NNLM in learning how best to work with public libraries in the future” [18]. The RMLs were an essential part of this project, assessing the public libraries in their areas that might be recruited for the study. Three RMLs—Middle Atlantic (Region 1), Southeastern Atlantic (Region 2), and South Central (Region 5)—participated, together with eight resource libraries and forty-one public libraries.

By design, the study started soon after NLM’s consumer health website, MedlinePlus, was launched in the summer of 1998. The RMLs provided equipment, promotional materials and onsite activities, and training for the public librarians. The pilot study participants also conducted outreach to communities of current or potential users, such as school and public health nurses, health clinic staff, disease-specific patient support groups, senior centers, employee groups, local hospitals, and church-based health centers. They helped sponsor health promotion and health-screening events, such as blood pressure tests, and did presentations about the project at meetings of library administrators, community leaders, and local government councils. While the activities generated only a modest increase in MedlinePlus traffic from library sites, the public librarians were enthusiastic about the training and new resources, and RML staff members began to learn about their new constituency [18].

Starting in 1995, NLM and NNLM professional outreach efforts expanded to include the public health workforce. That year, a group of public health professionals, medical informaticians, and medical librarians met to develop strategies for expanding public health use of advanced information technology. To address the need to improve information service to the diverse public health workforce, “NLM and NNLM joined forces with the Centers for Disease Control and Prevention (CDC), the Health Resources and Services Administration, the Association of State and Territorial Health Officials, the National Association of County and City Health Officials, and the Public Health Foundation to form Partners in Information Access for Public Health Professionals” [19, 20], now the Partners in Information Access for the Public Health Workforce (Partners). The Partners would collaborate to improve electronic resources useful in public health practice, increase awareness of existing resources, train public health professionals in the use of electronic information services, and help public health agencies obtain the equipment and Internet connections needed to use these services effectively.

In the first few years, the partnership carried out a two-year research project (1998–2000) that gathered and analyzed data on public health workers’ information needs, launched a website, and held a public health forum at NLM in 2001. NNLM staff served on the steering committees and helped shape many of the projects; they also contributed their substantial expertise to designing and maintaining the Partners website [19, 20]. This participation in the Partners initiative continued during the next fifteen years, leading to more NNLM public health outreach and the establishment of its National Public Health Coordinating Office in 2016 with the New England (Region 8) RML.

Outreach to Native Americans also became a priority for NLM in the late 1990s. Most of the RML regions included Native American communities, but their general outreach efforts had not yet specifically addressed the high levels of health disparities and lack of communication infrastructure in those areas. The Tribal Connections Project, which began in 1998 with the Pacific Northwest (Region 6) RML, aimed to establish or improve Internet connectivity, provide training, and build partnerships between the tribes and health education institutions.

The project was later extended to the Pacific Southwest (Region 7), MidContinental (Region 4), Greater Midwest (Region 3), and South Central (Region 5) RMLs [21, 22]. Importantly, the initial Pacific Northwest Region (Region 6) experience led to the publication of an outreach evaluation field manual, *Measuring the Difference: A Guide to Planning and Evaluating Health Information Outreach* (NNLM, Pacific Northwest Region, and NLM, 2000), and to the establishment of NNLM’s Outreach Evaluation Resource Center (OERC) several years later, which is still a gold standard for evaluation. Building on this work, the NNLM Pacific Northwest Region staff wrote a supplemental series of three booklets, *Planning and Evaluating Health Information Outreach Projects,* in 2006, and a second edition was published in 2013.

## THE NATIONAL NETWORK OF LIBRARIES OF MEDICINE IN THE NEW MILLENNIUM

The priorities of the late 1990s were reflected in the 2001–2006 RML contracts. The mission had shifted again, and the goals included the new focus on improving *public* access to health information. The RMLs were to work “with the NNLM and other organizations to increase public awareness of and access to health information via the Internet, with particular focus on senior citizens, minority populations, and persons of low socioeconomic status” [23]. Included in the section on outreach to health professionals was outreach to local and state public health departments, especially for Internet access and collaborations with public health organizations and institutions. “Consumer Health Information Services” was second on the outreach programs list, and this item emphasized working with other organizations, developing special programs for minorities, seniors, and others, and programs to promote MedlinePlus [23]. Under this contract, the RMLs jointly developed national goals and strategies for reaching public health departments and public libraries, using the public health “logic model” approach.

Starting in 2000, each RML’s staff included a consumer outreach coordinator. Extra funds were added to the 2001 to 2006 contracts for consumer health projects. The RMLs carried out a wide range of health information projects, partnering not just with public libraries, but with public health clinics, state health departments, senior centers, high schools, and charitable foundations (to name only a few). They reached out to public health nurses, school nurses, paramedics, HIV/AIDS caseworkers, and other care providers.

With assistance from the RML consumer health coordinators, NLM developed four consumer health courses for public librarians: “Prescription for Success: Consumer Health Information on the Internet,” “From Snake Oil to Penicillin: Looking in All the Wrong Places,” “PubMed for Public Librarians,” and “Beyond an Apple a Day: Providing Consumer Health Information in the Public Library.” These courses served as the nucleus for the Consumer Health Information Specialization offered by the Medical Library Association (MLA). They continue to be updated and taught by the RMLs. The success of all these efforts helped to further validate the NLM programs targeting the general public.

The 2001 to 2006 RML budget also established three new NNLM Centers. The NNLM National Training Center and Clearinghouse (NTCC), based at the New York Academy of Medicine (Region 1), would work with NLM to provide librarian training in databases like PubMed; collect information about training courses and web-based training materials produced by NLM, the RMLs, and NNLM members; and make such materials available for use in other settings. The NTCC is now the NNLM Training Office (NTO), based at the University of Utah’s Spencer S. Eccles Health Sciences Library. In coordination with NLM and the RMLs, the NTO delivers high-quality training to diverse audiences nationwide to support the effective use of NLM products and services. The NTO also ensures the efficient and effective development, delivery, and sharing of NNLM learning materials and training activities.

The NNLM OERC, based at the University of Washington (Region 6), would provide training and consulting services throughout NNLM and help NLM and RML network staff members design methods for measuring the effectiveness of overall network programs and individual outreach projects. The OERC is now the NNLM Evaluation Office (NEO), still based at the University of Washington’s Health Sciences Library.

The NNLM National Outreach Mapping Center (NOMC), based at Indiana University (Region 3) from 2001 to 2006, would help NLM and NNLM display the geographic distribution and impact of NNLM programs and services and to identify gaps that should be addressed. Outreach maps proved to be a valuable graphic tool for showing agency heads and Congress how much work was being done.

Since 2006, the variety of work carried out by the RMLs has continued to expand, still with emphasis on eliminating health disparities through outreach to underserved groups of health professionals, educators, patients, and consumers. NNLM staff have continued to develop online resources and make innovative use of new mobile technologies. To support the rapidly growing NNLM online presence, the network established the Web Services and Technology Operations Center (now the NNLM Web Services Office), which provides ongoing technical management of the network’s websites and investigates, recommends, and directs the implementation of additional web technology for teleconferencing, web broadcasting, distance education, and other activities.

Increasing concern about terrorist attacks and natural disasters after 2001 led federal and state agencies to add disaster planning to their twenty-first-century goals. NNLM had already demonstrated its value in disaster situations: in 2005, the RMLs in the Southeastern/Atlantic Region (Region 2) and South Central Region (Region 5) provided outstanding support for public health infrastructure in areas affected by Hurricanes Katrina and Rita, enabling delivery of needed information to displaced health professionals and others via public libraries and community organizations [24].

The new emphasis on emergency preparedness was added to the mission in the 2006 to 2011 RML contracts. In 2008, NNLM drafted the NNLM National Emergency Preparedness and Response Plan, a strategy for communicating among members and the RML offices before and during an emergency, and supporting network members with essential services. The plan led to the development of an online toolkit for use by network libraries in preparing for and responding to emergencies, as well as a tabletop exercise to test the plan and visits to each RML to conduct emergency preparedness and response training [25]. The network also implemented the RML Buddy System in the 2011 to 2016 contract, so that each RML would serve as a backup for another in emergencies, and all the RMLs would serve as backups for NLM customer service inquiries.

The 2011 to 2016 RML contract statement explicitly acknowledged the ever-changing landscape of health sciences library work and the need to adjust services to reflect new realities. It noted that digital technologies have transformed traditional resource sharing, from electronic publishing to collection development and access. With the proliferation of electronic journals, large-scale availability of journal back files, and major electronic collections, information seekers increasingly expect instant and free access; and social networking tools are promoting open access to knowledge and knowledge creation. Additions to the new contract thus included:

- allowing (for the first time) cross-regional collaboration on projects to expand RML services, when appropriate
- addressing the changing role of librarians and developing programs to assist librarians in promoting evidence-based health information and information management at their institutions
- participating in efforts to retain and preserve print materials, and to identify historical or unique materials in medicine and biomedical science, and increasing access to them, which included gathering information about history of medicine repositories in their regions for NLM’s *Directory of History of Medicine Collections*
- expanding outreach activities to vocational schools and community colleges, and to veterans, among others
- developing pilot projects to identify and promote the role of libraries in institutions that host electronic science initiatives

## A FIELD FORCE EXTRAORDINAIRE AND ITS CHALLENGES

It is easy to chronicle NNLM’s growth and accomplishments during its second generation and to marvel at the sheer number of projects carried out and the new directions taken. It is more difficult to convey, in an overview, the extent to which the network’s librarians have adapted to rapid changes, innovated, and collaborated to expand their remarkable and unique library network. The ever-expanding NNLM scope has posed diverse challenges for its staff.

One major challenge, which could be called “leaving the silo,” was described by Betsy L. Humphreys, FMLA, in her 2001 Janet Doe Lecture, “Adjusting to Progress: Interactions between the National Library of Medicine and Health Sciences Librarians, 1961–2001.” She noted that the early years of the RMLs demanded many adjustments: the implementation of the network “required academic health sciences librarians to work with people and institutions they had never worked with before, to adhere to policies they might not agree with, to serve users outside their customary clienteles, and—maybe worst of all—to make changes in internal procedures” [26]. Humphreys also noted that when outreach work was added to the RML work after 1990, the “field force” was not always happy to take on the job. Some medical librarians welcomed the mandate to do more outreach; others, though longtime users of NLM products and services, felt threatened (on several levels) by services designed for direct use by professionals, such as GRATEFUL MED and LOANSOME DOC, and feared that outreach duties would further erode their traditional role and divert resources from basic services [26].

The outreach initiatives required RML staff to step outside the traditional “comfort zone” of library work in a number of ways. Though they were accustomed to the “learning curve” (and slow connections) that went with using earlier computer systems, librarians were still challenged to master various new digital technologies and develop new ways to use them, especially during that early Internet era when connections often failed, small Internet service providers came and went, dial-up access was excruciatingly slow and often expensive, and software and servers did not always work well together. They also found that many people, both professional and nonprofessional, were confused by the jargon of the web universe.

Probably the greatest challenge overall was convincing nonlibrarians of the value of the growing cornucopia of health information resources. Missionaries of any sort often face a steep learning curve as they seek to bring benefits to other cultures. They cannot just assume that the “wonderfulness” of their offering is obvious—especially when there is a new language or a whole new paradigm to learn. Thus, RML staff found that many physicians and other health professionals only slowly embraced evidence-based medicine (which relies on easy access to current medical literature); they had little time to learn new skills and tended to draw on their basic medical training, their clinical experience, and their knowledge from occasional continuing education courses. Even some librarians saw little need to upgrade their skills and learn to use digital resources. Early efforts to connect hospital libraries to the Internet often met resistance from hospital administrators, who did not really understand what the Internet was or worried about costs and data security, and from hospital computer system staff, who did not want to collaborate.

Reaching out to public libraries, public health agencies, tribal communities, and community organizations took medical librarians a few more steps outside their traditional sphere. They could make even fewer assumptions and had to learn about the organizational structures, identify key leaders, and listen carefully to the expressed needs and concerns. Planning and evaluating outreach to such groups has been another major challenge for both NNLM and NLM. They have had to develop the skills of social scientists and cultural translators, as well as those of information professionals.

Overall, the collaborative network has grown wider and denser as the RMLs have partnered with other agencies, institutions, and organizations. NNLM currently includes over 6,000 full and affiliate members. The NLM *In Focus* series “Regional Medical Libraries Making a Difference” (2013–2015) featuring the individual RMLs shows how broad their outreach projects have become [27–34]. In recent years, for example, network staff have:

- trained librarians in research data management and systematic reviews of research data to aid with queries in evidence-based medicine
- educated hospital librarians to advocate for their libraries as essential components of hospital health care (e.g., the Middle Atlantic [Region 1] RML staff helping to develop a “Running Your Hospital Library Like a Business” six-hour course and the Greater Midwest Region [Region 3] RML’s “Measuring What Matters to Stakeholders” class)
- provided emergency preparedness training (e.g., producing YouTube videos and funding *Community Day*, a pilot project to assist libraries in becoming active partners in their community’s emergency preparedness, response, and recovery planning; RMLs in the Southeastern/Atlantic Region [Region 2], South Central Region [Region 5], and New England Region [Region 8] sponsored the pilots in their regions)
- provided back-up information support services to libraries affected by Superstorm Sandy in 2012
- supported a 6-year outreach project to public librarians in rural Tennessee, which helped 250 of them get MLA certification as Consumer Health Information Specialists
- helped Indian Health Service hospital staff develop relevant curricula on suicide awareness and prevention
- worked with the Rural Medical Service’s (RMS’s) Migrant Health Program to provide health education resources via the RMS clinics
- conducted health literacy workshops with community-based organizations to teach members how to better communicate with health care providers (e.g., a project with Kentucky Health Literacy for the Community, in the Greater Midwest Region)
- developed a huge number of consumer health websites and consumer health “toolkits” (e.g., *Finding Health and Wellness @ the Library: A Consumer Health Toolkit for Library Staff*, developed in a partnership between the California State Library and the NNLM Pacific Southwest RML [Region 7]; and the *Public Libraries and Community Partners* guide, developed to encourage partnerships between public libraries, community organizations, and network libraries)
- conducted outreach activities in primary and secondary schools
- continued outreach to Native American communities, tribal libraries, and tribal colleges, and to the health care providers who serve them, building on the *Tribal Connections* initiatives developed in the Pacific Northwest Region (Region 6), Pacific Southwest Region (Region 7), MidContinental Region (Region 4), and South Central Region (Region 5) RMLs and the OERC
- helped librarians and patrons to navigate the Affordable Care Act provisions and sign up for insurance
- increased access to multilingual health information
- helped assess and address the health information needs of underserved military families, particularly those including lesbian, gay, bisexual, or transgender individuals
- supported a community health information project at the Ma-Flo Salon in Georgetown, South Carolina, wherein the Southeastern/Atlantic Region (Region 2) staff trained the beauty salon staff to find health information on the web, who, in turn, helped their customers find health information, using computers located in the salon, and the salon’s owner, Marilynn Lance-Robb, has since gone out into the community to help train others to access health information

All these activities have been carried on in addition to those related to the core RML work described in the beginning of this article. DOCLINE and related services have remained a key part of RML responsibilities, though ILL requests have declined as more full-text articles become available online. Staff also have had to train with, use, and evaluate dozens of new NLM resources during the recent “digital explosion” and navigate the increasingly complex world of copyright and journal licensing, and the growing number of electronic journal resources. Also, they have in turn educated medical library professionals about newer tools, such as LinkOut for Libraries, to make the best use of literature resources.

The story of NNLM since 1985 is certainly a story about the vast expansion of information technology and the burgeoning online health information resources it supports. It is also the equally remarkable story of how medical librarians have reinvented their profession as they have explored and mapped the digital landscape, and trained others to do so. Their efforts have changed the way both professionals and the general public think about health information and its role in quality health care, and they continue to support and shape new roles for librarians as informationists in the evolving field of data science.

## SUPPLEMENTAL FILES

Appendix A National Network of Libraries of Medicine (NNLM) regions

Appendix B National Network of Libraries of Medicine (NNLM) Regional Medical Library (RML) directors and associate directors, 1985–2017

## References

[1] Bunting A (1987). The nation’s health information network: history of the Regional Medical Library Program, 1965–1985. Bull Med Libr Assoc.

[b2-jmla-106-162] 2An act to amend the Public Health Service Act to provide for a program of grants to assist in meeting the need for adequate medical library services and facilities, Pub. L. 89–291 (22 Oct 1965).

[3] Woodsmall RM (1988). MEDLINE on CD-ROM: National Library of Medicine evaluation forum.

[4] Lindberg DA, Siegel ER, Rapp BA, Wallingford KT, Wilson SR (1993). Use of MEDLINE by physicians for clinical problem solving. JAMA.

[b5-jmla-106-162] 5H.J.Res.395 - a joint resolution making further continuing appropriations for the fiscal year 1988, and for other purposes, Pub. L. 100–202, sec. 215 (22 Dec 1987).

[6] DeBakey ME, National Library of Medicine (US) (1989). Improving health professionals’ access to information: challenges and opportunities for the National Library of Medicine.

[b7-jmla-106-162] 7Request for contract action [memo]. 27 Oct 1989. Attachment C: statement of work. Located at: History of Medicine Division, National Library of Medicine, Bethesda, MD; Acc. 2012–002, NLM/RML/NNO; box 5, folder RFCA 1989.

[8] Wallingford KT, Ruffin AB, Ginter KA, Spann ML, Johnson FE, Dutcher GA, Mehnert R, Nash DL, Bridgers JW, Lyon BJ, Siegel ER, Roderer NK (1996). Outreach activities of the National Library of Medicine: a five-year review. Bull Med Libr Assoc.

[9] National Network of Libraries of Medicine MidContinental Region and Culver & Associates (1993). NN/LM-MR marketing research study: research findings.

[b10-jmla-106-162] 10RML directors meeting: minutes; Bethesda, MD; 16 May 1992. Located at: National Network Coordinating Office, National Library of Medicine, Bethesda, MD.

[b11-jmla-106-162] 11High-performance computing act of 1991, Pub-L 102–194 (9 Dec 1991).

[12] National Library of Medicine Annual report for fiscal year 1992.

[13] Rauch S, Holt MC, Horner M, Rambo N (1994). Community hospitals and the Internet: lessons from pilot connections. Bull Med Libr Assoc.

[14] Lacroix EM, Backus JE, Lyon BJ (1994). Service providers and users discover the Internet. Bull Med Libr Assoc.

[15] US National Library of Medicine 1999 follow-up survey of Internet connectivity in NNLM member libraries [Internet].

[b16-jmla-106-162] 16Statement of work for RML contract award period 1996–2001. Located at: National Network Coordinating Office, National Library of Medicine, Bethesda, MD.

[17] Deering MJ, Harris J (1996). Consumer health information demand and delivery: implications for libraries. Bull Med Libr Assoc.

[18] Wood FB, Lyon B, Schell MB, Kitendaugh P, Cid VH, Siegel ER (2000). Public library consumer health information pilot project: results of a National Library of Medicine evaluation. Bull Med Libr Assoc.

[19] Humphreys BL, Ruffin AB, Cahn MA, Rambo N (1999). Powerful connections for public health: the National Library of Medicine and the National Network of Libraries of Medicine. Am J Public Health.

[20] Cahn MA, Austin I, Selden CR, Cogdill K, Baker S, Cavanaugh D, Elliott S, Foster AJ, Leep CJ, Perez DJ, Pomietto BR (2007). The Partners in Information Access for the Public Health Workforce: a collaboration to improve and protect the public’s health, 1995–2006. J Med Libr Assoc.

[21] Wood FB, Sahali R, Press N, Burroughs C, Mala TA, Siegel ER, Fuller SS, Rambo N (2003). Tribal Connections health information outreach: results, evaluation, and challenges. J Med Libr Assoc.

[22] Siegel ER, Wood FB, Dutcher GA, Ruffin A, Logan RA, Scott JC (2005). Assessment of the National Library of Medicine’s health disparities plan: a focus on Native American outreach. J Med Libr Assoc.

[b23-jmla-106-162] 23Statement of work for RML contract period 2001–2006 (4 Feb 2000). Located at: National Network Coordinating Office, National Library of Medicine, Bethesda, MD.

[24] Cogdill KW, Ruffin AB, Stavri PZ (2007). The National Network of Libraries of Medicine’s outreach to the public health workforce: 2001–2006. J Med Libr Assoc.

[25] National Library of Medicine Annual report for fiscal year 2008.

[26] Humphreys BL (2002). Adjusting to progress: interactions between the National Library of Medicine and health sciences librarians, 1961–2001. J Med Libr Assoc.

[27] (2013). Regional Medical Libraries making a difference: focus on New England Region. NLM in Focus [Internet].

[28] (2013). Regional Medical Libraries making a difference: focus on Pacific Northwest Region. NLM in Focus [Internet].

[29] (2013). Regional Medical Libraries making a difference: focus on Pacific Southwest Region. NLM in Focus [Internet].

[30] (2014). Regional Medical Libraries making a difference: focus on South Central Region. NLM in Focus [Internet].

[31] (2015). Regional Medical Libraries making a difference: focus on Southeastern/Atlantic Region. NLM in Focus [Internet].

[32] (2015). Regional Medical Libraries making a difference: focus on the Middle Atlantic Region. NLM in Focus [Internet].

[33] (2014). Regional Medical Libraries making a difference: focus on Greater Midwest Region. NLM in Focus [Internet].

[34] (2014). Regional Medical Libraries making a difference: focus on MidContinental Region. NLM in Focus [Internet].

