# Supplementary material for: An historical overview of the National Network of Libraries of Medicine, 1985–2015
Source: J Med Libr Assoc. 2018 Apr 1;106(2):162–74. doi: 10.5195/jmla.2018.297 (PMC5886499; doi:10.5195/jmla.2018.297)

Journal of the Medical Library Association

Supplemental content to
J Med Libr Assoc. Apr;106(2):dx.doi.org/10.5195/jmla.2018.297
www.jmla.mlanet.org
© Speaker 2018

An historical overview of the National Network of Libraries of Medicine, 1985–2015

Susan L. Speaker, PhD

APPENDIX A

National Network of Libraries of Medicine (NNLM) regions

1. Middle Atlantic Region (MAR)
2. Southeastern/Atlantic Region (SEA)
3. Greater Midwest Region (GMR)
4. MidContinental Region (MCR)
5. South Central Region (SCR)
6. Pacific Northwest Region (PNR)
7. Pacific Southwest Region (PSR)
8. New England Region (NER)

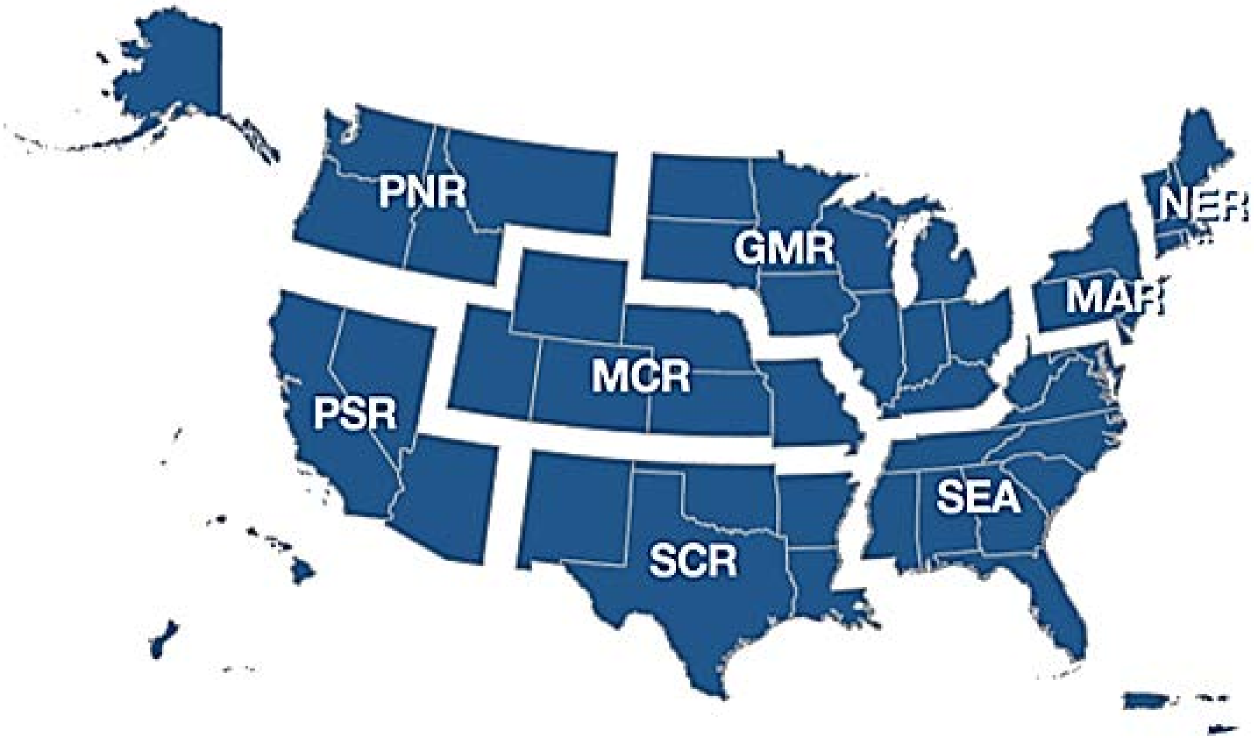

Supplement: Appendix A [file jmla-106-162-s001.pdf]
