# Supplementary material for: An historical overview of the National Network of Libraries of Medicine, 1985–2015
Source: J Med Libr Assoc. 2018 Apr 1;106(2):162–74. doi: 10.5195/jmla.2018.297 (PMC5886499; doi:10.5195/jmla.2018.297)
Supplement: Appendix B [file jmla-106-162-s002.pdf]

JMLA Journal of the Medical Library Association

Supplemental content to
J Med Libr Assoc. Apr;106(2):dx.doi.org/10.5195/jmla.2018.297
www.jmla.mlanet.org
© Speaker 2018

An historical overview of the National Network of Libraries of Medicine, 1985–2015

Susan L. Speaker, PhD

APPENDIX B

National Network of Libraries of Medicine (NNLM) Regional Medical Library (RML) directors and associate directors, 1985–2017

Directors and associate directors are listed in chronological order.

Region 1: Middle Atlantic Region

New York Academy of Medicine (1985–2006)

__Directors__

Brett Kirkpatrick
Arthur Downing
David King
Maxine Rocko

__Associate Directors__

Kay Mills Due
Mary Mylenki
Naomi Adelman

New York University (2006–2011)

__Directors__

Karen Brewer, FMLA
Neil Rambo

__Associate Directors__

Kathel Dunn
Kathleen Burr Oliver

University of Pittsburgh (2011–present)

__Director__

Barbara A. Epstein, AHIP, FMLA

__Executive Director__

Renae Barger

Region 2: Southeastern/Atlantic Region

University of Maryland (1985–present)

__Directors__

Cyril Feng (deceased)
Frieda O. Weise, FMLA
Mary Joan (MJ) Tooey, AHIP, FMLA

Supplemental content to
J Med Libr Assoc. Apr;106(2):dx.doi.org/10.5195/jmla.2018.297
www.jmla.mlanet.org
© Speaker 2018

Executive Directors

Carol G. Jenkins, AHIP, FMLA
Suzanne F. Grefsheim, FMLA
Faith A. Meakin, AHIP, FMLA
Janice E. Kelly, FMLA
James Dale Prince, AHIP

Region 3: Greater Midwest Region

University of Illinois, Chicago (1985–2016)

Directors

Frieda O. Weise, FMLA
Elaine Russo Martin
Susan Jacobson, AHIP
Kathryn (Kate) Carpenter

Associate Directors

Ruby May
Jan Ahrensfeld
Janice E. Kelly, FMLA
Linda Walton, AHIP
Jean Sayre (deceased)
Ruth Holst, AHIP, FMLA
Beth A. Layton, AHIP

University of Iowa (2016–present)

Director

Linda Walton, AHIP

Associate Director

Elizabeth (Liz) Kiscaden, AHIP

Region 4: MidContinental Region

University of Nebraska (1985–2001)

Directors

Robert Braude, AHIP, FMLA
Nancy Woelfl

Associate Directors

Elizabeth (Betty) Petgen
Richard Pride
Carolyn Reed
Dorothy Willis
Deborah Ward
Peggy Mullaly-Quijas, AHIP
Rebecca (Becky) Satterthwaite

University of Utah (2001–present)

Directors

Wayne J. Peay, FMLA
Jean Shipman, AHIP, FMLA

Associate Director

Claire Hamasu

Region 5: South Central Region

University of Texas Southwestern Medical Center (1985–1991)

Director

Jean Miller (deceased)

Associate Directors

John (Pat) Murphey (deceased)
James (Pat) Craig
Regina Lee

Houston Academy of Medicine-Texas Medical Center Library (1991–2016)

Directors

Richard (Dick) A. Lyders, AHIP, FMLA
Naomi C. Broering, AHIP, FMLA
J. Robert (Bob) Beck
Elizabeth (Liz) Eaton (deceased)
L. Maximilian (Max) Buja

Executive/Associate Directors

Mary Ryan
Annanaomi Sams
Renée Bougard
Michelle Malizia
Owen Ellard (interim)
Jon Goodell, AHIP

University of North Texas Health Science Center (2016–present)

Director

Daniel (Dan) Burgard

Executive Director

Lisa Smith

Region 6: Pacific Northwest Region

University of Washington (1985–present)

Directors

Gerald (Gerry) Oppenheimer
Sherrilynne Fuller, FMLA
Tania P. Bardyn, AHIP

JMLA Journal of the Medical Library Association

SC
Supplemental content to
J Med Libr Assoc. Apr;106(2):dx.doi.org/10.5195/jmla.2018.297
www.jmla.mlanet.org
© Speaker 2018

Associate Directors

Dale Middleton
Neil Rambo
Catherine (Cathy) Burroughs

Region 7: Pacific Southwest Region

University of California, Los Angeles (1985–present)

Directors

Alison Bunting, AHIP, FMLA
Judy Consales

Associate Directors

Darcy Van Vuren
Elaine Graham
Beryl Glitz, FMLA
Elaine Graham
Heidi Sandstrom
Julie Kwan, AHIP
Alan F. Carr, AHIP

Region 8: New England Region

University of Connecticut (1985–2001)

Director

Ralph Arcari

Associate Directors

Linda Walton, AHIP
John Stey

University of Massachusetts (2001–present)

Director

Elaine Russo Martin

Associate Directors

Deborah (Debbie) Sibley
Javier Crespo
Mary Piorun, AHIP
